# Embarking on improvement cycles - the next steps in surgical benchmarking

**DOI:** 10.1007/s00423-025-03860-z

**Published:** 2025-10-10

**Authors:** Michaela Ramser, Fariba Abbassi, Matthias Pfister, Franca Bergfelder, Milo A. Puhan, Matthias Turina

**Affiliations:** 1 Department of Visceral Surgery and Transplantation, University Hospital Zurich, Rämistrasse 100, Zurich, 8091 Switzerland; 2 Epidemiology, Biostatistics and Prevention Institute, University of Zurich, Zurich, Switzerland; 3 University of Zurich, Wyss Zurich Translational Center, Zurich, Switzerland

**Keywords:** Benchmarking, Quality improvement, Improvement cycle, Statistical process control, Moving median, Low anterior resection, Rectal cancer, Outcome, Colorectal

## Abstract

**Purpose:**

To develop a standardized, clinically applicable methodology for comparing surgical outcomes with established benchmark cut-offs and guiding structured quality improvement. Benchmarking compares clinical outcomes with defined performance thresholds to identify areas for improvement. While benchmark values are available for various surgical procedures, there is no standardized methodology that allows for direct application in clinical practice. This limits their use in routine quality management and continuous improvement processes.

**Methods:**

A structured quality improvement cycle was developed to compare own data with surgical benchmark cut-offs for ideal (and non-ideal patients). The approach includes periodic comparison of clinical outcomes with benchmark cut-offs, root cause analysis for deviations, and implementation of targeted interventions. The method was applied to a cohort of 98 patients undergoing low anterior resections between 2018 and 2023. Outcomes were analyzed over overlapping 18-month rolling windows, updated every 6 months, to track trends and assess adherence to benchmarks.

**Results:**

The analysis revealed deviations from benchmark targets, especially in readmission rates due to ileostomy-related complications. Root cause analysis identified gaps in postoperative care and patient education. In response, targeted measures were implemented, including multimedia-based ostomy education, improved nutrition protocols, enhanced outpatient support, and structured follow-up. These interventions aim to reduce deviations and improve outcomes in future assessment cycles.

**Conclusions:**

This methodology allows comparison of surgical outcomes with established benchmark cut-offs to guide structured quality improvement. It enables surgical teams to identify outcome gaps, implement data-driven interventions, and foster continuous quality improvement. The framework is adaptable to various procedures with existing benchmarks and promotes evidence-based surgical excellence.

**Supplementary Information:**

The online version contains supplementary material available at 10.1007/s00423-025-03860-z.

## Introduction

Benchmarking is a method of comparing results against a standardised set of parameters that have been recognized as best practice [1]. Originally introduced in manufacturing and business management, benchmarking has since been successfully adopted in surgical outcome research [2–4]. The methodology for establishing surgical benchmarks, specific values for important outcomes that reflect high but achievable surgical quality, was validated in a Delphi process [5] and has since been published for a number of surgical procedures like major liver surgery [6], transthoracic esophagectomy [7], liver transplantation [8], robotic distal pancreatectomy [9] and many more [10–22].

However, there is currently no methodology available to integrate benchmarks into a quality improvement cycle to effectively improve clinical practice.

In surgical benchmarking, performance cut-offs (benchmarks) are defined as the 75th/25th percentile of all analysed reference centre medians of a given outcome parameter and for a predefined sub-population of ‘ideal’ patients, i.e. low-risk patients [1]. For example, in our previous study of low anterior resection (LAR) for elective rectal cancer surgery, the median anastomotic leak rate was calculated for all 19 contributing centres separately and the benchmark was deliberately set at the (achievable) 75th percentile of all centres medians (benchmark cut-off for anastomotic leak rate of 9.8% for ideal patients; range of 0-15.6%) [19].

The idea of benchmarking is to compare one’s own data or that of centres, registries and regions with established benchmark cut-offs. This process helps identify quality gaps and encourages the implementation of targeted improvement measures.

The methodology presented herein enables surgeons and hospitals to compare their data against established benchmarks, derive meaningful insights, and implement targeted improvement initiatives within their departments, where appropriate. Importantly, this approach is not limited to LAR but is broadly applicable to any surgical procedure for which benchmark values exist. As part of a continuously recurring quality improvement cycle, this methodology supports the enhancement of both patient outcomes and process quality.

## Methods

### Consensus development and methodological framework

The authors, a multidisciplinary team of professionals in surgery, medical outcome research and epidemiology met for several consensus meetings to define the methodology for outcome comparison with published benchmark cut-offs. The benchmark study of LAR for rectal cancer was selected as the reference procedure for developing this framework.

Three key requirements were identified as essential for the methodology:

- Continuous quality assessment: short time periods for analysis to allow for prompt detection of trends and deviations.
- Adequate sample size: To ensure statistical robustness and prevent distortion by outliers, a sufficient number of patients needed to be grouped together.
- A straightforward calculation method: The analytical approach had to be feasible for repeated use without excessive workload.

Respecting these requirements, we selected the moving median of any given parameter of interest (such as length of hospital stay (LOS)) as the preferred statistical variable. The moving median serves a purpose similar to the moving average, which is used in various forms in statistics, macroeconomic analysis and finance and is an established method for smoothing short-term fluctuations [23]. And was therefore deemed statistically more appropriate due to its robustness against outliers and skewed distributions applicable for continuous variables such as duration of surgery (in minutes), Comprehensive Complication Index (CCI), or length of hospital stay (LOS), calculated over overlapping 18-month periods. For categorical variables such as anastomotic leakage or readmission rates, a moving median is not applicable. Instead, we calculated the event rate within each defined period, resulting in a moving rate that similarly tracks trends over time while respecting the nature of the data.

### Determination of optimal time intervals for analysis

**Fig. 1:**
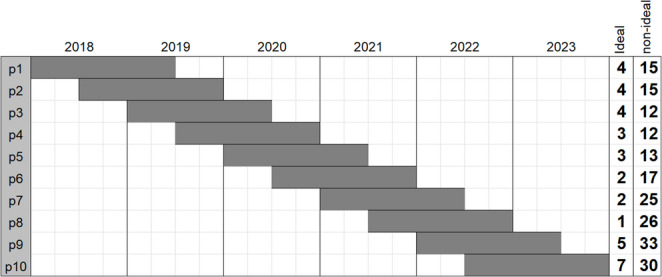
Overlapping 18-months time windows in a continuous analysis. Overlapping 18-month rolling time windows (dark grey) were used to ensure a sufficient number of patients per period (p1-p10) for the analysis and comparison of periodic outcome data (moving median/rate) against the benchmark cut-offs

To strike a balance between timely intervals and sufficient case accumulation, various times intervals of analysis were tested for their effect on smoothing fluctuations and minimizing outlier influence. Finally, we defined the half-yearly evaluation with a rolling window of 18-months to ensure a uniform presentation of the data (Fig. 1).

### Primary and secondary outcome

Primary outcome of this analysis was the establishment of a methodology to compare own clinical data with established benchmark cut-offs.

As secondary outcome the methodology was tested at our own patient population and all steps of the benchmarking cycle explained. We therefore analysed prospectively collected data from patients undergoing resection of low and mid rectal adenocarcinoma at our institution from January 2018 to December 2023 securing a follow-up of at least 6 months. The analysis was finalised in December 2024, leading to the next steps of the improvement cycle.

### Data collection and inclusion criteria

All analyses were performed using prospectively collected data from patients undergoing resection of low and mid rectal adenocarcinoma at our institution from January 2018 to December 2023 with raw data sourced from the AQC database (“Arbeitsgemeinschaft für Qualitätssicherung in den Chirurgischen Disziplinen”). The inclusion and exclusion criteria were identical to the characteristics defined in our previously published benchmark study [19]. Briefly, all patients undergoing elective LAR for an adenocarcinoma of the rectum independent of the surgical approach (robotic, laparoscopic or open) or reconstruction (with or without anastomosis). Abdominoperineal resections were excluded from this analysis.

Patients were stratified according to the given definition into ideal and non-ideal groups following the established benchmark for LAR [19]. Ideal patients were therefore defined as aged ≥ 18 to < 80 years, BMI ≥ 20 to < 35 kg/m2 without previous major abdominal surgery, a preoperative albumin of ≥ 3.0 g/dL, no recent history of smoking, ASA score < 3, no anticoagulation, no known bleeding disorder, no chronic obstructive lung disease, no insulin-dependent diabetes, no steroid use or immunosuppressive therapy, no IBD, TNM stage pT ≤ 3, no preoperative chemotherapy.

This division allows for a more homogeneous group to be compared, reduces confounding caused by comorbidities or other risk factors, enables meaningful risk-adjusted comparisons, and facilitates the identification of trends across different patient cohorts.

### Ethical approval

Only patients with a signed general consent for the routine use of administrative data were included. Ethical approval for this analysis was obtained from the Canton Zurich, Switzerland (BASEC 2024-00510).

## Results

**Fig. 2:**
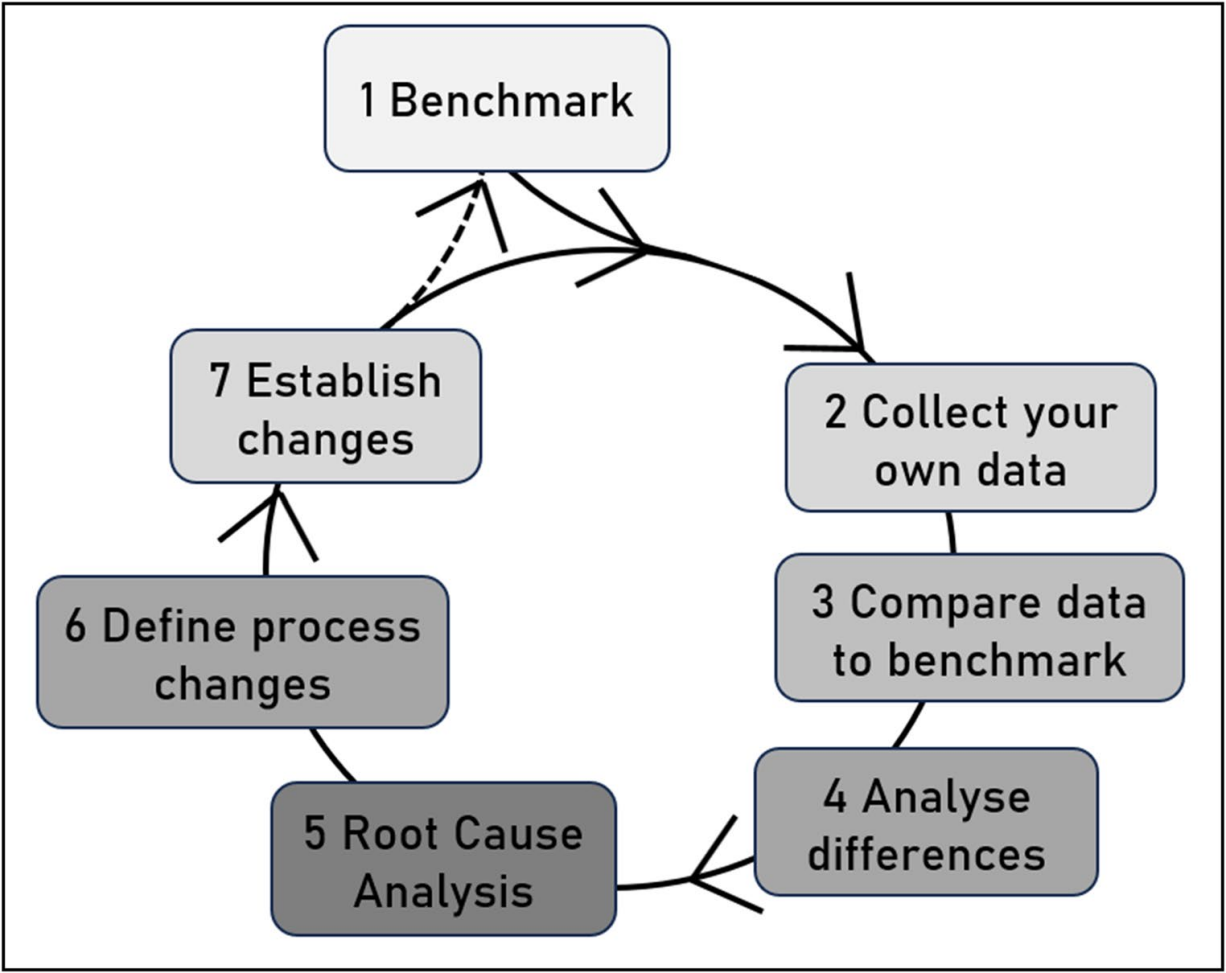
The quality improvement cycle of surgical benchmarking. The quality improvement cycle consists of seven steps, with steps two through seven detailed in this project. This project references an existing benchmark trial, using established benchmark cut-offs for data comparison and analysis of differences. Clinically relevant deviations should be thoroughly analysed, leading to a review of standard operating procedures and discussions on necessary process adaptations. The final step focuses on change management, ensuring the implementation of procedures and strategies to enhance quality and outcomes

The data-driven improvement cycle requires the establishment of benchmark cut-offs as *step 1* (Fig. 2). For rectal cancer resections, these cut-off values were published by our group in 2022 based on outcome data of international reference centres [19].

### Collect your own data – step 2

A total of 98 patients were treated with an elective LAR between January 2018 to December 2023 and included in our analysis, with a follow-up period of six months extending through June 2024. According to the criteria defining the two patient groups, 16 patients (16.3%) were identified as ideal patients. Baseline characteristics are shown in Tables 1 and 2.

**Table 1:** Patient’s characteristics

	Overall, *N* = 98	Ideal patients, *N* = 16	Non-ideal patients, *N* = 82
Age (years) (median; IQR)	66 (56, 75)	58 (53, 69)	67 (57, 76)
Gender (n; %)			
Female	34 (35%)	4 (25%)	30 (37%)
Male	64 (65%)	12 (75%)	52 (63%)
BMI (kg/m2) (median; IQR)	24.3 (21.9, 28.4)	25.3 (23.0, 28.5)	23.9 (21.9, 28.4)
ASA score (n; %)			
0	1 (1.0%)	0 (0%)	1 (1.2%)
1	1 (1.0%)	0 (0%)	1 (1.2%)
2	48 (49%)	16 (100%)	32 (39%)
3	46 (47%)	0 (0%)	46 (56%)
4	2 (2.0%)	0 (0%)	2 (2.4%)
Congestive heart failure (n; %)	6 (6.1%)	0 (0%)	6 (7.3%)
Myocardial infarction (n; %)	5 (5.1%)	0 (0%)	5 (6.1%)
Atrial fibrillation (n; %)	8 (8.3%)	0 (0%)	8 (10%)
Vascular comorbidity (n; %)	12 (12%)	0 (0%)	12 (15%)
Chronic kidney disease (n; %)	7 (7.2%)	0 (0%)	7 (8.6%)
COPD (n; %)	4 (4.1%)	0 (0%)	4 (4.9%)
Diabetes (n; %)	3 (3.1%)	0 (0%)	3 (3.7%)
Hypoalbuminemia (n; %)	6 (6.1%)	0 (0%)	6 (7.3%)
Immunosuppression (n; %)	4 (4.1%)	0 (0%)	4 (4.9%)
Smoking (n; %)	22 (22%)	0 (0%)	22 (27%)
Anticoagulation (n; %)	13 (13%)	0 (0%)	13 (16%)
History of major abdominal surgery (n; %)	16 (16%)	0 (0%)	16 (20%)
Local tumor stage (yp, p) (n; %)			
T0	8 (8.2%)	0 (0%)	8 (9.8%)
Tis	2 (2.0%)	0 (0%)	2 (2.4%)
T1	18 (18%)	5 (31%)	13 (16%)
T2	26 (27%)	4 (25%)	22 (27%)
T3	37 (38%)	7 (44%)	30 (37%)
T4	7 (7.1%)	0 (0%)	7 (8.5%)
Nodal status (n; %)			
N0	65 (66%)	7 (44%)	58 (71%)
N1	26 (27%)	6 (38%)	20 (24%)
N2	7 (7.1%)	3 (19%)	4 (4.9%)
Metastatic disease (n; %)	12 (13%)	1 (6.3%)	11 (14%)
No. of resected LN (median; IQR)	21 (16, 26)	22 (21, 24)	20 (16, 26)
≥ 12 lymph nodes resected (n; %)	94 (96%)	15 (94%)	79 (96%)
R0 resection (n; %)	94 (98%)	15 (94%)	79 (99%)
Distance from anal verge (n; %)			
lower third (0–6 cm)	22 (22%)	3 (19%)	19 (23%)
middle third (7–12 cm)	76 (78%)	13 (81%)	63 (77%)
Surgical approach (n; %)			
open	13 (13%)	0 (0%)	13 (16%)
laparoscopic	10 (10%)	2 (13%)	8 (9.8%)
robotic	75 (77%)	14 (88%)	61 (74%)
Conversion to open (n; %)	7 (7.1%)	1 (6.3%)	6 (7.3%)
Duration of surgery (min) (median; IQR)	339 (299, 368)	313 (290, 345)	343 (300, 371)
Primary anastomosis (n; %)	84 (86%)	16 (100%)	68 (83%)
Ostomy type (n; %)			
definitive colostomy	14 (14%)	0 (0%)	14 (17%)
ileostomy	84 (86%)	16 (100%)	68 (83%)

^1^Median (IQR); n (%)

*BMI* body mass index; *ASA* American Society of Anesthesiologists; *COPD* chronic obstructive pulmonary disease; *LN* lymph nodes; *IQR* interquartile range

**Table 2:** Outcome data

	Overall, *N* = 98	Ideal patients, *N* = 16	Non-ideal patients, *N* = 82
Postoperative LOS (d) (median; IQR)	8 (6, 12)	7 (5, 8)	8 (6, 14)
Intermediate care (IMC) (n; %)	6 (6.1%)	0 (0%)	6 (7.3%)
IMC LOS (d) (median; IQR)	1 (1, 1)	-	1 (1, 1)
Intensive care (ICU) (n; %)	6 (6.1%)	0 (0%)	6 (7.3%)
ICU LOS (d) (median; IQR)	2 (1, 3)	-	2 (1, 3)
Anastomotic leak (n; %)	8 (8.2%)	0 (0%)	8 (9.8%)
Clavien-Dindo (n; %)			
2	1 (13%)	-	1 (13%)
3a	5 (63%)	-	5 (63%)
3b	2 (25%)	-	2 (25%)
At discharge			
Clavien-Dindo ≥ 3a (n; %)	15 (15%)	0 (0%)	15 (18%)
CCI (median; IQR)	9 (0, 22)/ 13 (15)	0 (0, 9)/ 7 (11)	9 (0, 23)/ 14 (16)
Mortality (n; %)	0 (0%)	0 (0%)	0 (0%)
Discharge to 3 months			
Clavien-Dindo ≥ 3a (n; %)	10 (10%)	4 (25%)	6 (7.3%)
CCI (median; IQR)	0 (0, 21)/ 9 (13)	9 (0, 27)/ 14 (16)	0 (0, 9)/ 8 (12)
Readmission (n; %)	19 (19%)	5 (31%)	14 (17%)
Mortality (n; %)	0 (0%)	0 (0%)	0 (0%)
At 3 months			
Clavien-Dindo ≥ 3a (n; %)	10 (10%)	4 (25%)	6 (7.3%)
CCI (median; IQR)	21 (0, 31)/ 19 (16)	16 (0, 27)/ 18 (18)	21 (9, 31)/ 20 (16)
Mortality (n; %)	0 (0%)	0 (0%)	0 (0%)
3 to 6 months			
Readmission (n; %)	1 (1.0%)	0 (0%)	1 (1.2%)
Mortality (n; %)	1 (1.0%)	0 (0%)	1 (1.2%)
At 6 months			
Mortality (n; %)	1 (1.0%)	0 (0%)	1 (1.2%)

Median (IQR); n (%)

*LOS* length of stay; *IMC* intermediate care; *ICU* intensive care unit; *CCI* comprehensive complication index; *IQR* interquartile range

### Compare data to benchmark – step 3

**Fig. 3:**
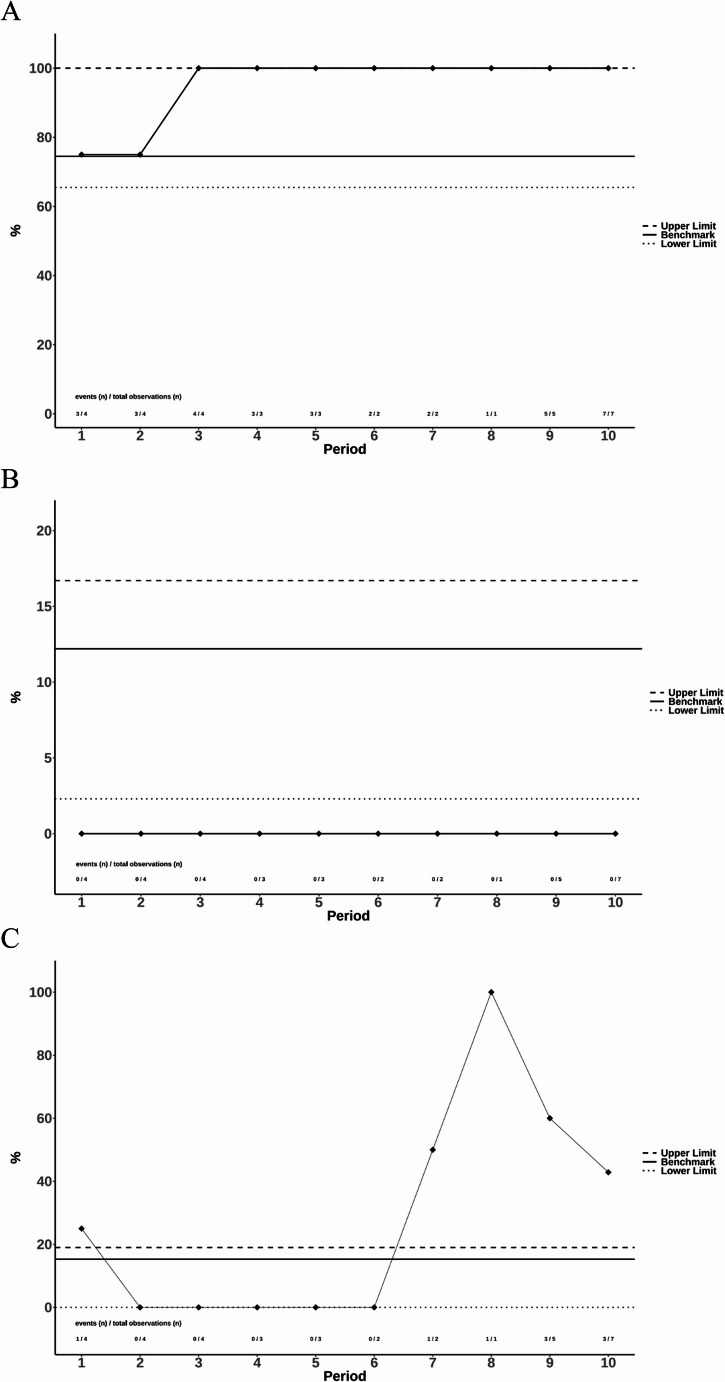
Compare data to benchmark. **A** ideal patients – rate of ≥ 12 lymph nodes. Lymph node retrieval: The rate of ≥ 12 lymph nodes was analysed for the 10 overlapping time windows for ideal patients (A) at six-month intervals, comparing the results with published benchmark cut-offs and ranges (upper and lower limits). All results remained above the benchmark (firm line), indicating consistently excellent results that do not require further investigation. **B** ideal patients - major complications (CDC ≥ 3a) at discharge. The rate for major complications (CDC ≥ 3a) at discharge was analysed for the 10 overlapping time windows for ideal patients (B) at six-month intervals, comparing the results with published benchmark cut-offs and ranges (dotted lines). In our cohort, no major complications occurred in ideal patients indicated as all analysed periods (black line) are located below the benchmark which is at 12.2%. **C** ideal patients - readmission rate. The readmission rate within the first three months after the index hospitalization was analysed for the 10 overlapping time windows for ideal patients (C) at six-month intervals. Results were compared with published benchmark cut-offs (firm line) and deviations (periods 7–10) were flagged for further analysis as part of step 4 of the improvement cycle

All outcome parameters with benchmark cut-offs were analysed for each overlapping 18-months period (total of 1o periods) and our results compared with the published benchmark and its upper and lower range. Below a selection of benchmark cut-offs and the comparison with our outcome data as graphics are shown (Fig. 3 and Supp 1)

The benchmark cut-off for the percentage of patients with a number of harvested lymph nodes ≥ 12 is 74.5% (range 65.5–100) in ideal patients and 68.3% (range 54.3–94.7) in non-ideal patients [19]. This means that 74.5% is the 75th percentile of all centre’s medians included in the original benchmark study. Therefore, results better than the cut-off, are within or above the benchmark. The lower and upper limit (dotted line) indicate the range of centres medians from the benchmark study [19]. The 10 intervals analysed from our data are shown as black line with dots (Fig. 3a and Supp 1a).

The benchmark cut-off for major complications at discharge (CDC ≥ 3a) in ideal patients is 12.2% (range 2.3–16.7) and 13.7% (range 3.3–26.0) for non-ideal patients [19]. Accordingly, our results represented in Fig. 3b and Supp 1b. Deviations from the benchmark, as observed for the rate in major complications in non-ideal patients in periods 1–4 (Supp 1b) have been highlighted for later analyses in step 4 of the improvement cycle.

The final example presented here is the readmission rate for the first three months after discharge of the index stay (Fig. 3c and Supp 1c). The benchmark cut-off for readmission rates is 15.3% (range 0.0–19.0) in ideal patients and 21.7% (range 3.2–28.6) in non-ideal patients [19]. Notable deviations from these benchmarks were observed in ideal patients during periods 7–10 and in non-ideal patients during periods 2 and 3. These deviations were flagged for further analysis as part of step 4 in the improvement cycle.

### Analyse differences – step 4

To investigate the flagged deviations, we conducted a more detailed analysis using the readmission rate as an example. Patients from the affected time periods were identified, and their clinical courses were reviewed to explore potential causes for the elevated readmission rates observed during those intervals.

We analysed periods 8–10 in ideal patients regarding readmission rate and found the following reasons for readmissions:

- Patient #1058: high-output ileostomy.
- Patient #1046: small bowel obstruction proximal to diverting loop ileostomy following a high-fibre meal.
- Patient #1066: combination of high-output ileostomy and ileus. Difficult ostomy education due to foreign language.

Reviewing the cases that resulted in a deviation from the benchmark, we found that all were due to ileostomy-related complications. We therefore concluded, that a root-cause analysis for stoma-related complications had to be performed as suggested in step 5 of the improvement cycle.

For non-ideal patients the following patients contributed to a deviation from the benchmark regarding readmission rate:

- Patient #1029: high output ileostomy.
- #1036: patient with cognitive impairment, social decompensation at home due to stoma.
- Patients #1048 and #1060: pain. Computed tomography shows a presacral collection. Endoscopy without anastomotic leak. Abscess drainage and antibiotic therapy.
- Patient #1069: diarrhoea and abdominal pain after ileostomy reversal. Congestive heart failure requiring monitoring and consultation with cardiology.
- Patient #1005: Renal failure in colostomy patient requiring IV resuscitation.

In conclusion, we observed different reasons for readmission within 3 months after the index operation in non-ideal patients comprising mostly ostomy-related morbidity as well as deep surgical site infections (SSI).

As only two patients were readmitted due to surgical site infections (SSI) over the entire five-year period, a root cause analysis was not recommended.

### Root cause analysis – step 5

**Fig. 4:**
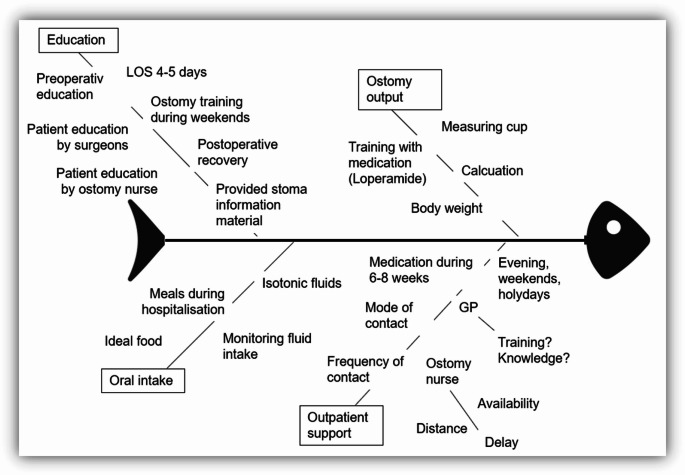
Root cause analysis of ileostomy-related readmissions

Root cause analyses are performed to detect the underlying problem in a process that shows deviation from the norm. The approach is used in different areas and has become part of frameworks and models like Lean Management, Total Quality Management (TQM). Here, we use a fish bone diagram as described by Kaoru Ishikawa within his work on Quality Control [24]. As an example, we used the results from the readmission rate for a root cause analysis with the question why stoma-related problems led to readmissions. After outlining patient pathways and processes before, during, and after hospitalization, the expert surgeons used a fishbone diagram to guide brainstorming and conduct a cause-and-effect analysis of potential ileostomy-related issues, incorporating input from ward nurses, ostomy care specialists, and surgical residents (Fig. 4). Four possible areas were identified, namely ostomy education, ostomy output management, control of oral intake and outpatient support. Ostomy counselling started only in the postoperative phase. During that time, patients recover from surgery, have issues with nausea or pain. Postoperative length of hospital stay in ideal patients was 5–8 days, mostly including a weekend as our OR days are typically Thursday and Friday. Therefore, ostomy nurse guided training is reduced to 2–3 days. Apart from stoma bag changing, all the other subjects have to be covered during this time (recommended nutrition, how to measure stoma output, calculating ideal stoma output, first measures if stoma-output is too high, etc.).

After discharge, patients are often left without specific ostomy support. General practitioners (GP) have no surgical training and therefore minimal stoma related knowledge. Patients have outpatient’s appointments with ostomy nurses but only irregularly as they need to travel to a hospital or look for outpatient ostomy care.

In the next step, possible interventions of change were defined.

### Define process change – step 6

Based on the results of *step 5*, the following measures were initiated throughout the year 2024. To enhance patient education and ensure 24/7 access to reliable information before and after surgery, we created a dedicated ostomy education video. Its purpose is to improve understanding of key topics such as ostomy output management and regulation of oral intake, while offering a consistent and streamlined educational resource. The video is now hosted on our institutional website, and patients receive a QR code linking directly to it. Patients are introduced to the video during their preoperative visit in the outpatient clinic, when surgical consent is obtained. This early access allows them to view the material as often as needed throughout the perioperative period, reinforcing comprehension and supporting self-care. The QR code linking to the video is also included in discharge letters, making it accessible to referring specialists and general practitioners. This aims to improve knowledge of ostomy management beyond the hospital setting and promote more consistent care in the outpatient environment.

Ostomy education provided by nursing staff and ostomy care nurses has been streamlined to ensure consistency with the content of our educational video.

To align postoperative meals with the nutritional guidance provided in our educational video, an improvement project on postoperative nutrition was initiated in collaboration with the nutritionist team. The goal was to establish a dedicated low-fibre “colorectal” postoperative diet consistent with our clinical recommendations. This new diet will address a key issue with the standard hospital menu, which frequently includes high-fibre meals that not only increase the risk of ostomy blockages but also confuse patients by contradicting the advice they receive from clinical staff (project ongoing).

As a recurring issue in ostomy output management was patients running out of prescriptions for psyllium or loperamide, we now routinely provide long-term prescriptions at the time of discharge valid for repeated use and covering at least three months.

The final improvement project, which is still ongoing, focuses on enhancing the outpatient pathway through closer patient follow-up either in person or by phone, ideally conducted by clinical nurses or ostomy care specialists.

### Establish change – step 7

The above-mentioned measurements are now introduced in daily practice and their effect will be measured during the next cycles of quality improvement.

## Discussion

We developed an algorithm that allows the comparison of prospectively collected data with benchmarks as part of a continuously repeating quality improvement cycle with the aim of enhancing patient outcomes and process quality.

The introduction of surgical benchmarking was an important step towards improving surgical quality and benchmark cut-offs were since published for several surgical procedures [6–19]. However, it is the methodology proposed in this study that enables the adoption of benchmarking in daily practice by comparing institutional data with the best achievable results.

The presented methodology aligns with well-established benchmarking frameworks used in manufacturing and business management, where systematic processes evaluate performance relative to industry leaders. This concept was first widely implemented in the automobile industry where companies such as Toyota pioneered the use of lean manufacturing and Kaizen (continuous improvement) principles to improve production efficiency and quality [25]. Therefore, understanding the problem to be solved is defined (plan), change carried out (do). Then, it is checked that change has led to the intended improvement and if so, the change is integrated into daily routine as new standard (PDCA: plan – do – check – act). This approach to problem solving promotes the continuous and systematic pursuit of improvement through getting continuously more and better knowledge about the processes. Similarly, Six Sigma, widely used in operations management and quality improvement, relies on statistical thresholds to reduce variability and defects [26] and Statistical Process Control (SPC) is a well-established methodology in operations research to monitor production processes, reducing variability and maintaining high-quality output [27, 28].

In healthcare and surgery, such principles are increasingly being recognized and applied in a similar manner [29–32]. By integrating continuous monitoring and real-time data analysis, our approach integrates many of the above listed methods and ensures that deviations in surgical outcome are promptly identified and addressed.

Given that the group of ideal patients is small in most centres and adverse outcome in this group are relatively rare, a statistically robust methodology was required. It needed to both detect systematic deviations in outcome and group enough patients and events for a meaningful interpretation. To address this, we used a rolling 18-month time window with a repetition of the analysis done every 6 months, the moving median.

The graphs employing moving medians should be interpreted with professional judgement, weighing the clinical relevance of individual events against the scale of deviation. Rare events in small populations inevitably generate large visual spikes, which, while unavoidable, serve nevertheless a purpose. Such events should remain visible in the overall analysis, and if they occur more frequently, they should prompt reflection and eventually action. To support this interpretation, selected principles from Statistical Process Control (SPC) can provide additional structure for distinguishing random variation (“noise”) from meaningful changes (“signals”). For instance, a repeated deviation above or below the defined benchmark over several consecutive periods may indicate a true signal rather than isolated variability. Although traditional SPC tools such as control limits are not applied in this benchmarking context, patterns of sustained deviation can still help guide further analysis and potential quality improvement measures, complementing clinical expertise with structured, data-informed interpretation.

For non-ideal patients, deviations from the benchmark often have diverse causes. In contrast to the ideal patients, the group of non-ideal patients is very inhomogeneous and can include patients with only one exclusion criteria (e.g. a 40-year insulin-dependent diabetic) as well as patients with multiple exclusion criteria (e.g. 85-year-old polymorbid patient with chronic obstructive pulmonary disease, immunosuppression, severe peripheral artery disease and chronic renal insufficiency). Identifying patterns in recurring complications and deviations from the benchmark within this group is therefore particularly valuable but must be interpreted with caution. Beyond baseline characteristics, treatment strategy further distinguishes this heterogeneous group. Non-ideal patients are frequently older, burdened by comorbidities, or carry an unfavourable risk profile. In such cases, surgical strategies differ substantially, for example by opting against reconstruction in favour of a stoma, thereby limiting the comparability of outcomes such as anastomotic leakage. Importantly, the stratification not only reflects patient baseline factors but also indirectly captures treatment strategy and surgical judgment, which themselves influence outcomes.

However, in our view, certain deviations from the benchmark do not necessarily indicate a quality issue. For instance, in our study population, the duration of surgery was constantly longer than the benchmark cut-off would suggest for ideal as well as non-ideal patients. Several factors could explain this, such as our preference for robotic surgery, which despite being performed by experienced robotic surgeons and with dual consoles, typically takes slightly longer than laparoscopic or open procedures [33]. Additionally, our clinic is a training hospital for surgeons, scrub nurses, anaesthetists, and nursing staff. Teams change daily, making process standardization more challenging. Therefore, we monitor duration of surgery but do not consider it a primary concern as long as deviations remain uniform and without significant outliers.

Similarly, conversion rate should not automatically be classified as a complication. Analysing our cases more in-depth, we found that conversions were not due to intraoperative complications such as uncontrollable bleeding or organ injury. Instead, conversions were often pre-emptively chosen for patient benefit, particularly in cases involving previous surgeries and extensive intraabdominal adhesions were confirmed using a minimally-invasive approach. Conversion was then considered less likely to cause complications such as small bowel injuries. As a result, our conversion rate analysis did not necessitate further steps, we therefore decided not proceed to step 5 of the improvement cycle which would have been a root cause analysis.

Another example is the rate of loop ileostomies created in LAR with TME. Rather than indicating complications or quality issues, this practice reflects a surgical strategy for anastomoses located within 8 cm of the anocutaneous junction especially after neoadjuvant chemoradiation. Importantly, our low rate of anastomotic insufficiency highlights the effectiveness of this approach, also seen in our ileostomy reversal rate of nearly 100%, indicating very few severe long-term anastomotic complications that would prevent stoma reversal.

In summary, the graphical representation using moving medians/rates to compare our results with benchmark cut-offs, confirmed intuitive assumptions about our surgical quality while presenting the data in an objective and easily comprehensible manner. This approach highlighted opportunities for discussion and initiated further analysis if needed.

The methodology presented here enables surgeons and hospitals to compare their data against the published benchmark, draw meaningful conclusions and implement improvement initiatives in their departments if indicated.

We recognize that high-quality surgical care is not limited to high-resource or high-volume centres. The quality improvement model and benchmarking approach presented here are designed to be inclusive and applicable across diverse healthcare settings. Benchmarks are established using a structured Delphi process that engages high-volume expert centres from various continents, ensuring that different healthcare systems, cultures, and resource levels are represented [1, 5]. By setting performance targets at the 75th or 25th percentile, the benchmarks are intentionally designed to be ambitious yet achievable, even for smaller or resource-limited institutions. Moreover, benchmarks have been developed not only for complex oncologic procedures such as low anterior resection, esophagectomy, gastrectomy, and perihilar cholangiocarcinoma surgery [7, 17, 19, 21], but also for non-oncologic interventions including primary and redo liver transplantation and robotic bariatric surgery [8, 18, 22]. This underscores the versatility and broad applicability of the benchmarking concept across a wide spectrum of surgical practice.

While implementing benchmarking cycles within one’s own clinic may initially require considerable effort, the knowledge gained about outcomes, complications, and results in comparison with high-volume centres nevertheless provides valuable returns. Depending on the baseline quality already achieved, the immediate clinical consequences of benchmarking cycles may not be substantial. However, in our view, the repeated and structured engagement with one’s own quality will increase awareness and help prevent unnoticed decline.

One limitation of our study concerns the overall case volume. While our inclusion criteria were deliberately strict and aligned with the benchmark study on LAR, the resulting sample size may appear modest. However, upon reviewing the original benchmark study, we note that even international high-volume centres contributed between 100 and 200 cases, with only a subset meeting the “ideal patient” criteria. Nevertheless, the applicability of our methodology may be limited in very low-volume centres. That said, LAR for rectal cancer is typically performed in specialised, high-volume institutions in most countries, which mitigates the relevance of this limitation in the intended context of use.

## Conclusion

This study presents a framework for integrating benchmarking into day-to-day clinical practice of surgical quality improvement, decision-making and patient management. By enabling the comparison of real institutional data against benchmarks, this approach seamlessly incorporates benchmarking into a continuous quality improvement cycle, refining surgical practices and enhancing patient outcomes over time. Notably, this methodology is applicable not only to LAR but also to all other surgical procedures with established benchmarks.

### Ethical approval

Only patients with a signed general consent for the routine use of administrative data were included. Ethical approval for this analysis was obtained from the Canton Zurich, Switzerland (BASEC 2024 − 00510).

### Statistical analysis

Statistical analyses were performed using Microsoft Excel (Microsoft, Redmond, WA, USA) and RStudio (R Foundation for Statistical Computing, Vienna, Austria). For each time period, the median or rate (for binary outcomes) of each outcome indicator was calculated and plotted against the corresponding benchmark values published by Staiger et al. [19].

## Supplementary Information

Below is the link to the electronic supplementary material.

Supplementary Material 1

## Data Availability

No datasets were generated or analysed during the current study.
