# Supplementary material for: Embarking on improvement cycles - the next steps in surgical benchmarking
Source: Langenbecks Arch Surg. 2025 Oct 10;410(1):298. doi: 10.1007/s00423-025-03860-z (PMC12513939; doi:10.1007/s00423-025-03860-z)
Supplement: Supplementary file 1 — Supplementary Material 1 [file 423_2025_3860_MOESM1_ESM.docx]

**Supp 1. Compare data to benchmark**

**1A non-ideal patients:** Lymph node retrieval


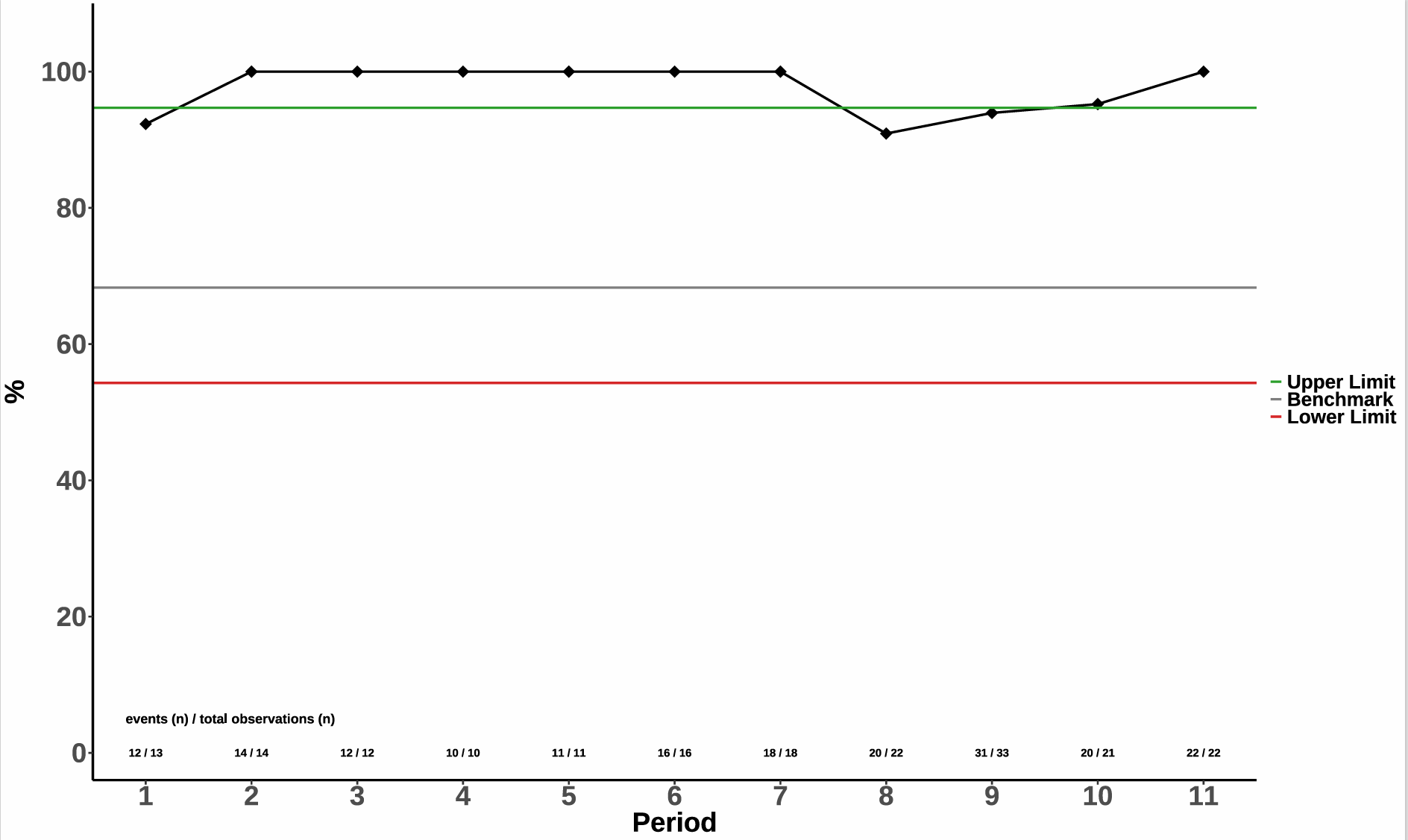


Lymph node retrieval: The rate of ≥12 lymph nodes was analysed for the 11 overlapping time windows for non-ideal patients at six-month intervals, comparing the results with published benchmark cut-offs and ranges (upper and lower limits). All results remained within the benchmark range (between the red and green lines).

**1B non-ideal patients:** Rate of major complications


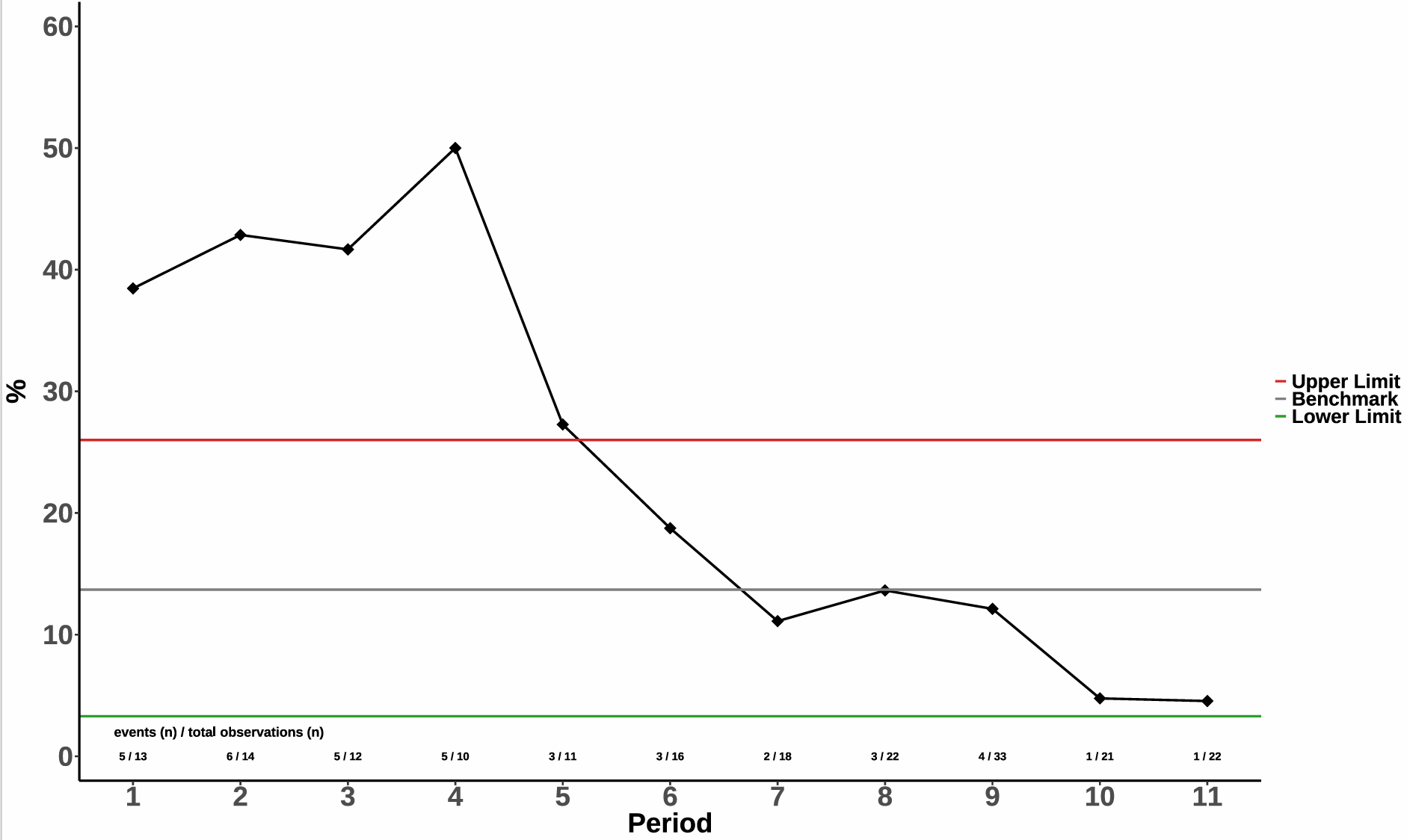


Rate of major complications: The rate for major complications (CDC ≥3a) at discharge was analysed for the 11 overlapping time windows for non-ideal patients at six-month intervals, comparing the results with published benchmark cut-offs and ranges (upper and lower limits). In our cohort, major complications occurred more frequently than the benchmark cut-off would suggest, necessitating in-depth analysis of periods 1–6.

**1C non-ideal patients:** Readmission rate


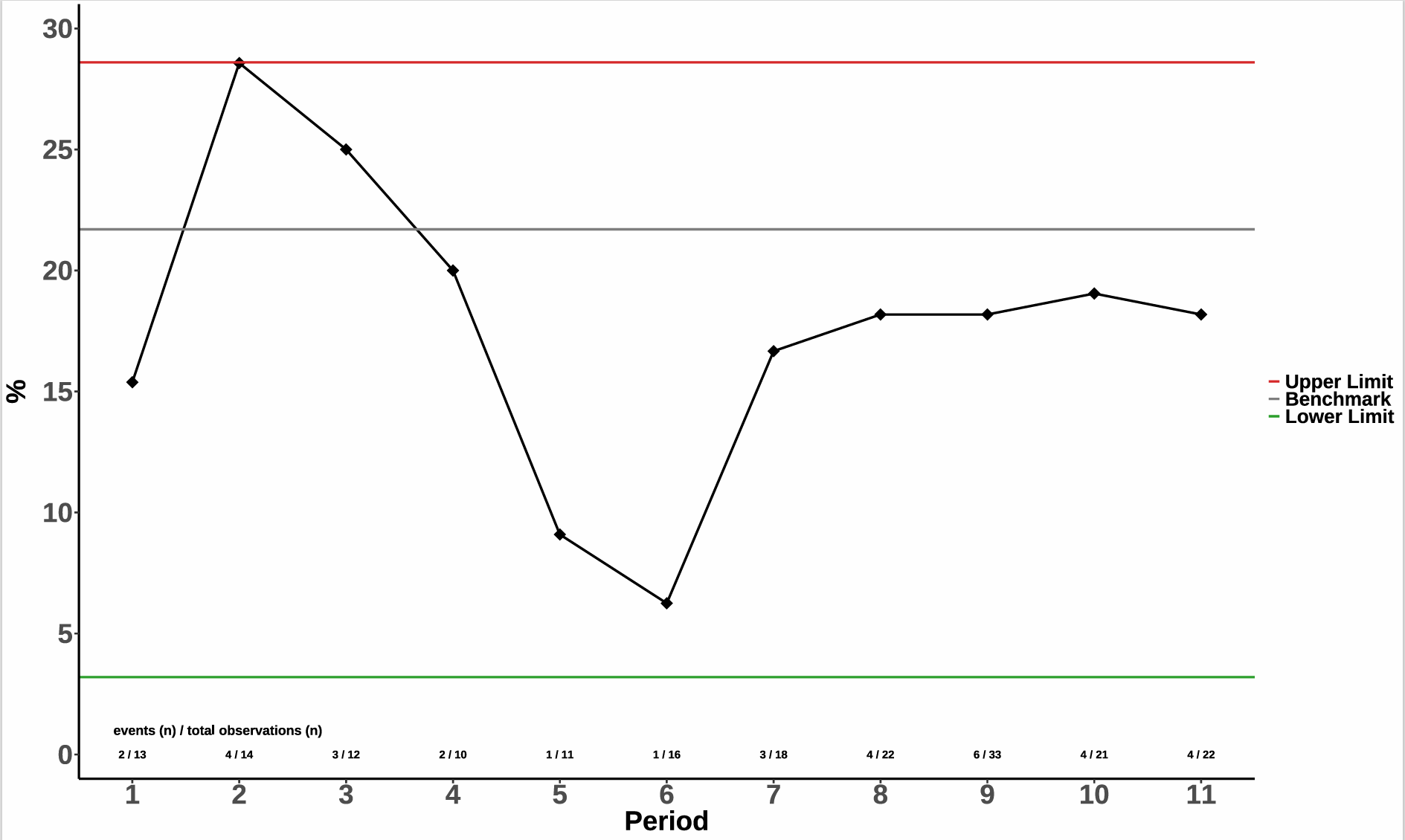


Readmission rate: The readmission rate within the first three months after the index hospitalization was analysed for the 11 overlapping time windows for non-ideal patients at six-month intervals. Results were compared with published benchmark cut-offs and the established range (upper and lower limits) and deviations were flagged for further analysis as part of step 4 in the improvement cycle.
